# Cold Hypersensitivity in the Hands and Feet May Be Associated with Functional Dyspepsia: Results of a Multicenter Survey Study

**DOI:** 10.1155/2016/8948690

**Published:** 2016-03-16

**Authors:** Kwang-Ho Bae, Ju Ah Lee, Ki-Hyun Park, Jong-Hyang Yoo, Youngseop Lee, Siwoo Lee

**Affiliations:** ^1^Mibyeong Research Center, Korea Institute of Oriental Medicine, 1672 Yuseongdae-ro, Yuseong-gu, Daejeon 305-811, Republic of Korea; ^2^KM Fundamental Research Division, Korea Institute of Oriental Medicine, 1672 Yuseongdae-ro, Yuseong-gu, Daejeon 305-811, Republic of Korea

## Abstract

*Aim*. To investigate whether dyspepsia symptoms differ depending on the presence or absence of cold hypersensitivity in the hands and feet (CHHF).* Methods*. In all, 6044 patients were recruited and provided with a questionnaire about CHHF and dyspepsia. Based on their responses, subjects were divided into a CHHF group (persons who noted cold sensations; *n* = 1209) and a non-CHHF group (persons who noted warm or intermediate sensations; *n* = 1744). The groups were compared in terms of their usual digestion status, using chi-square tests and logistic regression analyses to calculate the propensity score and odds ratios (ORs). We analyzed the participants' responses to questions on dyspepsia symptoms.* Results*. After matching, chi-square tests indicated that the CHHF group had higher frequencies of the following symptoms: bad digestion, poor appetite, discomfort in the upper abdomen, motion sickness, epigastric burning, postprandial fullness, nausea, and bloating. Additionally, CHHF was associated with an increased OR for dyspepsia (bad digestion, vomiting, motion sickness, epigastric burning, postprandial fullness, nausea, epigastric pain, and bloating) compared with the non-CHHF group.* Conclusion*. This study confirmed that CHHF patients have elevated frequencies of most dyspepsia symptoms.

## 1. Introduction

In traditional Korean medicine, pattern identification is important to both understanding the patient's status and writing a prescription. There are several methods of pattern identification, including 8-principle pattern identification, constitutional pattern identification, and visceral pattern identification [1, 2]. The 8-principle system includes the following factors: yin and yang, exterior and interior, cold and heat, and deficiency and excess. Cold and heat is an important factor for revealing the patient's status. In Korean medicine, cold and heat do not refer to the patient's body temperature alone but instead are an inclusive concept that incorporates the patient's subjective feeling of warmth and chill, as well as fevers diagnosed by a doctor (4 examinations). Cold and heat is a phenomenon that appears when functional activities decline or increase due to diseases or constitution.

Because cold and heat pattern identification is diagnosed based on both the patient's symptoms and 4 examinations by a doctor of Korean medicine, objective evidence is necessarily insufficient for diagnosis. Further, evidence on the effects of cold and heat status on the body also remains uncertain. To resolve these issues, recent studies have attempted to provide objective standards for cold and heat, including the development of a cold pattern questionnaire by Ryu et al. [3] and a study in which Song et al. [4] investigated the dependence of Korean medicine prescriptions on cold and heat disposition in knee osteoarthritis. However, studies of cold and heat and the effects of their status on the body have been performed rarely to date. The rarity of such studies may be explained by the absence of verified diagnostic tools for cold and heat pattern identification; there is considerable uncertainty when conducting a study of cold and heat with relatively broad inclusion criteria and an open-ended definition of the pattern.

Therefore, we decided to conduct the present study using a narrower range of inclusion criteria and a more specific scope of research. First, rather than investigating the dual states of cold and heat, we limited our investigation to differences between cold and noncold individuals. Second, rather than recruiting subjects based on various symptoms of coldness, we specifically analyzed cold hypersensitivity in the hands and feet (CHHF), which is a representative cold symptom. CHHF is relatively common symptom in Korea and is more frequently observed in women than in men [5]. Patients with CHHF feel coldness both in cold places and at temperatures that are relatively warm. The prevalence rate of CHHF is somewhat uncertain because there is insufficient data to obtain an accurate estimate; however, 38.7% of women complained of coldness in a study by Kondo and Okamura [6]. Third, to investigate the effects of cold on body function, we specifically investigated differences in digestive function between the CHHF and non-CHHF groups via functional dyspepsia symptoms. Our decision to investigate digestive function was based on the relatively high prevalence rate of functional dyspepsia (8–30%) [7] and the “spleen and 4 extremities” theory of the* Huangdi Neijing* [2], which states that the limbs are connected to the spleen. This theory implies that digestive function affects the 4 limb extremities, meaning that the limbs are healthy when the digestive function is healthy and diseased when the digestive function is poor.

Previous studies have also reported that cold hypersensitivity and dyspepsia are correlated [8, 9]. However, those studies targeted limited subjects such as women or patients of a specific age group or involved an insufficient sample size. Thus, additional studies are necessary to clearly demonstrate this relationship. With these considerations in mind, we hypothesized that individuals with CHHF would have a poorer digestion status than those without CHHF. Accordingly, we investigated differences in digestion status among persons with and without CHHF by analyzing responses to a questionnaire.

## 2. Methods

### 2.1. Data Collection

**Figure 1:**
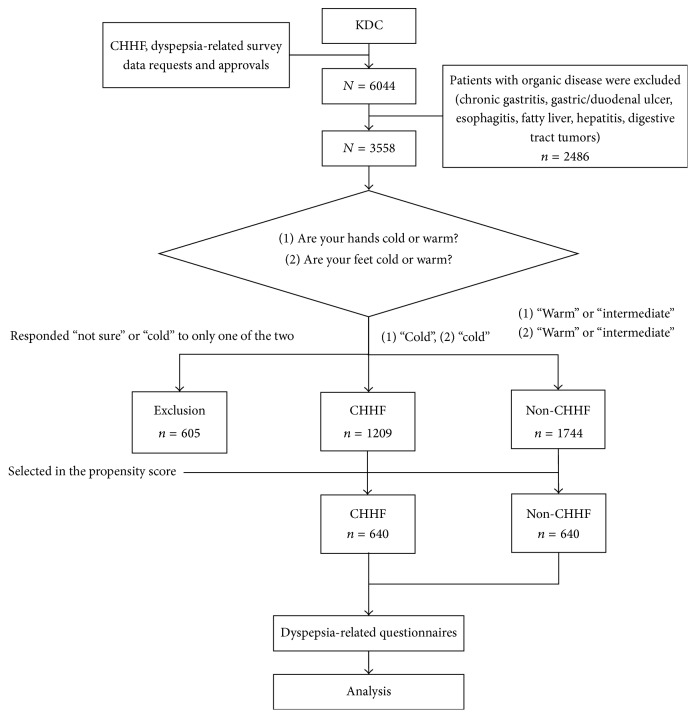
Flow chart of the study. KDC: Korean Medicine Data Center; CHHF: cold hypersensitivity in the hands and feet; non-CHHF: noncold hypersensitivity in the hands and feet.

This cross-sectional study was conducted between November 2006 and August 2014. All of the questionnaire data, including CHHF and dyspepsia status, were compiled from the Korean Medicine Data Center (KDC) of the Korea Institute of Oriental Medicine (KIOM) [10]. Using this resource, we collected questionnaire data on 6044 adults (19 years old or older) who were admitted to 13 traditional Korean medicine hospitals and 11 traditional Korean medicine clinics. To isolate our analysis from any effects of organic dyspepsia, we excluded data on patients diagnosed with chronic gastritis, gastroduodenal ulcers, esophagitis, fatty liver, hepatitis, or digestive tract tumors. After applying these exclusions, 3558 individuals remained. Among them, patients were excluded who showed unclear symptoms for classification into the CHHF group or non-CHHF group. The remaining 2953 individuals were selected as the final study subjects, including 1209 persons in the CHHF group and 1744 persons in the non-CHHF group (Figure 1). This study was approved by the Institutional Review Board of KIOM (I-0910/02-001).

### 2.2. Cold Hypersensitivity in the Hands and Feet

Those who responded “cold” to the question “are your hands cold or warm?” and those who responded “cold” to the question “are your feet cold or warm?” were classified as the CHHF group. Those who responded “warm” or “normal” to the both of these questions were classified as the non-CHHF group. We excluded those who stated that they were cold in response to only 1 of these 2 questions because the presence of CHHF symptoms appeared to be unclear. For similar reasons, we excluded those who stated that they were unsure in response to either question.

### 2.3. Questionnaire on Digestion

The questionnaire included 9 items that refer to common complaints in Korea and were derived from descriptions in the Rome II classification [11]. In addition to these 9 items, the questionnaire included items on 3 topics that are needed to apply pattern identification in Korean medicine: digestion status (“how is your digestion?”), motion sickness, and exhaustion when hungry. The definition for each symptom was based on the description presented in Rome II [12]. The subjects were asked to answer the questionnaire based on their usual status within the past 6 months, which was chosen because the Glasgow Dyspepsia Severity Score [13] includes evaluations of symptoms during the latest 6 months and because the same symptom duration was presented in the most recent Rome III classification.

The details of each question were as follows. To the question “how is your digestion?” the subjects chose either “1. good” or “2. bad.” To the question “how is your appetite?” and an item related to anorexia, the subjects chose “1. very good,” “2. good,” “3. average,” or “4. not good.” The criteria for these responses were as follows: “1. very good” refers to the desire to eat more foods despite satiety after meal; “2. good” refers to the case in which one feels hungry at mealtimes and wants to eat food; “3. average” refers to the case in which one eats meals at mealtimes but does not have a good appetite; and “4. not good” refers to the case in which one has no appetite at mealtimes and does not have a good sense of taste, even when eating. For the items on dyspepsia symptoms (discomfort in the upper abdomen, vomiting, motion sickness, exhaustion when hungry, belching, epigastric burning, postprandial fullness, nausea, epigastric pain, and abdominal bloating), respondents were asked to choose 1 of the following answers: “1. often,” “2. sometimes,” and “3. rarely.” “1. often” refers to greater than or equal to 2 times per week, “2. sometimes” refers to greater than or equal to 3 times per month, and “3. rarely” refers to less than or equal to 2 times per month (Supplementary Table 1; see Supplementary Material available online at http://dx.doi.org/10.1155/2016/8948690).

### 2.4. Statistical Analysis

**Figure 2:**
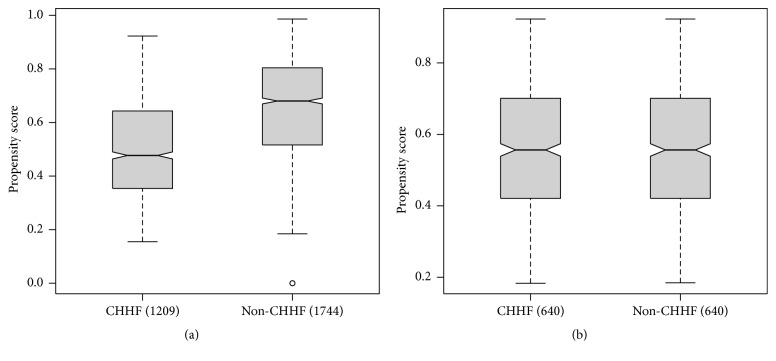
Comparison of the propensity score between the CHHF group and non-CHHF group before and after propensity matching. (a) Propensity score before matching; (b) propensity score after matching. CHHF: cold hypersensitivity in the hands and feet; non-CHHF: noncold hypersensitivity in the hands and feet.

The statistical program SPSS 21.0 for Windows (IBM Corp., Armonk, NY, USA) was used for statistical analysis. The general characteristics of the subjects were matched using a propensity score consisting of sex, age, and BMI, with the matching process involving a minimum distance scoring method.  Figure 2 shows the alteration in propensity score distribution between the matched CHHF and non-CHHF groups. These physical characteristics were presented as frequencies and percentages or means ± standard deviations. Between-group comparisons were performed using the chi-square test (for categorical variables) and the independent-samples *t*-test (for continuous variables). The chi-square test was used to analyze the frequencies and percentages of responses to digestion-related questions in the CHHF and non-CHHF groups. In addition, logistic regression was performed to calculate the odds ratios (ORs) for dyspepsia in the propensity-matched group as well as in the original groups. The OR was determined for each dyspepsia-related item in the CHHF group compared to the non-CHHF group. The statistical significance level was set at *P* < 0.05.

## 3. Results

### 3.1. Demographic Characteristics

The number of subjects in the original CHHF and non-CHHF groups was 1209 and 1744, respectively. The total study sample included more women (*n* = 1958; 66.3%) than men (*n* = 995; 33.7%). The female-to-male ratio was much higher in the CHHF group (983 women, 81.4%, versus 226 men, 18.7%) than in the total study sample. The mean ages in the CHHF and non-CHHF groups were 44.6 and 47.4 years, respectively. The mean height and weight in the non-CHHF group were 1.7 cm taller and 6.8 kg heavier, respectively, than the corresponding values in the CHHF group. The mean BMIs in the CHHF and non-CHHF groups were 22.0 and 24.1, respectively. The general characteristics were significantly different between CHHF and non-CHHF groups before matching (all *P* < 0.001). After propensity score matching, the total number of patients in each group was 640, with no statistically significant differences in general characteristics between the groups (Table 1).

### 3.2. Chi-Square Tests of the Relationship between CHHF and Dyspepsia

Before matching, the CHHF group and non-CHHF group significantly differed (*P* < 0.001) in every dyspepsia item. A higher proportion of the CHHF group reported bad digestion and not good appetite compared to the non-CHHF group, and the frequency of all dyspepsia symptoms (discomfort in the upper abdomen, vomiting, motion sickness, exhaustion when hungry, belching, epigastric burning, postprandial fullness, nausea, epigastric pain, and bloating) was also higher.

After matching, significant differences were detected in digestion, postprandial fullness, bloating (*P* < 0.001), discomfort in the upper abdomen, motion sickness, epigastric burning, postprandial fullness, nausea, and appetite (*P* < 0.05). The frequency of dyspepsia symptoms was higher in the CHHF group compared to the non-CHHF group, while there was no statistically significant difference between the two groups in items of vomiting, exhaustion when hungry, belching, and epigastric pain (related to digestion) (Table 2).

### 3.3. The Odds Ratios for Dyspepsia according to CHHF Status

As can be seen in Table 3 and Supplementary Figure 1, ORs were used to investigate the differences in dyspepsia between the CHHF and non-CHHF groups. Before propensity matching, a significant between-group difference was observed for all items.

After matching analyses, bad digestion, motion sickness, postprandial fullness, bloating (*P* < 0.001), vomiting, epigastric burning, nausea, and epigastric pain (*P* < 0.05) significantly differed between the groups, and there was no significant difference for not good appetite, discomfort in the upper abdomen, exhaustion when hungry, and belching. The OR was highest for bad digestion (2.423) before matching and for bloating after matching (1.883) (Table 3).

For the OR of each response (“often” and “sometimes”) for dyspepsia symptoms after matching, both responses (“often” and “sometimes”) for bloating significantly differed between groups. There was a significant difference in either “often” or “sometimes” responses for discomfort in the upper abdomen, vomiting, motion sickness, exhaustion when hungry, epigastric burning, postprandial fullness, nausea, and epigastric pain, and there was no difference in belching (Supplementary Figure 1).

## 4. Discussion

We chose to investigate the relationship between CHHF (including various cold symptoms) and indigestion because of the “spleen and 4 extremities” theory that was described in Huangdi's classic text [2], which is one of the most famous works in oriental medicine. As stated in this theory, the spleen is included in the human digestive system and controls the passage of nutrition to the 4 limbs, and therefore symptoms in the 4 extremities are thought to relate to the function of the spleen. This theory is taken into consideration when prescribing acupuncture or herbal medicine in the clinic. Furthermore, because the prevalence of functional dyspepsia is high (8–30%), we expected that it would be relatively easy to investigate correlations between CHHF and dyspepsia [7].

CHHF refers to a condition in which one experiences discomfort in daily living because of cold symptoms in the limbs. It is more inclusive than Raynaud's phenomenon and includes decreased temperature in the hands and feet, as well as the subjective sensation of cold. CHHF is suspected to induce spastic peripheral vasoconstriction, but no specific, certain cause has yet been identified. In Korea, CHHF is a relatively common symptom and occurs more often in women than in men [5]. The diagnosis and treatment of CHHF are a relatively active topic of research in Korea, including recent studies by Park et al. [14] and Hur et al. [5]. In another study of the relationship between CHHF and diseases, Tokunaga et al. [15] investigated “Hie,” which refers to oversensitivity to coldness. Kondo and Okamura [6] also investigated the relationship between CHHF and the Cornell Medical Index (CMI).

Dyspepsia is a digestive function disorder that refers to the collective symptoms of the upper gastrointestinal tract. In general, the term “dyspepsia” denotes functional dyspepsia; in this study, we therefore excluded persons who had been diagnosed with chronic gastritis, gastroduodenal ulcers, esophagitis, fatty liver, hepatitis, and digestive tract tumors. Through these exclusions, we sought to remove as many cases of organic dyspepsia as was feasible. In Rome III [16], functional dyspepsia is defined as the presence of symptoms including postprandial satiety, early satiety, gastric pain, and epigastric burning without any organic disease. Although the Rome III definition is generally accepted, the diagnostic standard of Rome III has not been applied strictly to many cases in clinical practice, and therefore the diagnostic period of dyspepsia has remained somewhat controversial [17].

In the present study, the data were compiled from questionnaire responses that had been collected by KDC. Participants who noted having cold hands and feet were assigned to the CHHF group, while participants who had neither cold hands nor cold feet were assigned to the non-CHHF group. Between-group differences in indigestion were analyzed using the KDC digestion questionnaire. Several recent studies have used the KDC data, including those by Do et al. [18] study on the Sasang constitutional diagnostic method, Chae et al. [19] on the development of the Sasang constitution questionnaire, and Jang et al. [20] on metabolic syndrome.

The aim of this study was to verify whether there was a difference in functional dyspepsia frequency according to the presence of CHHF and, if so, which of the various digestion-related symptoms differed. In summary, patients with CHHF showed a high frequency of dyspepsia, with this tendency generally maintained after matching. Bloating in particular significantly differed between groups both before and after matching: the highest OR after matching was 1.883 and the responses “often” and “sometimes” were significantly more common in the CHHF group.

The general characteristics of subjects differed markedly depending on the presence of CHHF. In the CHHF group, the female-to-male ratio (81%) was much higher compared to the non-CHHF group (56%), and the mean age and BMI were higher in the non-CHHF group (47.4 years and 24 kg/m^2^ versus 44.6 years and 22 kg/m^2^, resp.). We concluded that these differences could harbor considerable bias in examining the relation between CHHF and dyspepsia. Therefore, the patients were matched in the CHHF group and non-CHHF group using the propensity score matching method (640 patients per matched group). There was no difference in sex, BMI, and age after matching. These differences in general characteristics confirmed that CHHF was influenced by sex and BMI, as in previous studies [5, 6].

This study suggests that there is a correlation between CHHF and functional dyspepsia. In the chi-squared test shown in Table 2, the CHHF group and non-CHHF group significantly differed in all items before matching and in digestion, appetite, discomfort in the upper abdomen, motion sickness, epigastric burning, postprandial fullness, nausea, and bloating after matching. There was no significant difference in vomiting, exhaustion when hungry, belching, or epigastric pain.

As shown in Table 3, the OR for the development of dyspepsia symptoms (bad digestion, not good appetite, and sum of “often” and “sometimes” for the occurrence of each symptom) was increased in every item before matching in the CHHF group, and a significantly increased OR was observed for bad digestion, vomiting, motion sickness, epigastric burning, postprandial fullness, nausea, epigastric pain, and bloating after matching. A significant OR was not observed for not good appetite, discomfort in the upper abdomen, exhaustion when hungry, or belching. These results revealed a slight difference in the items with significant differences according to the analysis method but generally supported a high frequency of dyspepsia in the CHHF group.

In this study, we observed a significant difference between the frequencies of indigestion in participants who did and did not have CHHF. Previously, Tokunaga et al. [15] found that symptom frequencies differed according to the presence of Hie (oversensitivity to coldness), and Nietert et al. [21] found that Raynaud's phenomenon was associated with undiagnosed vascular disease. Together with these earlier investigations, the present study provides evidence supporting the notion that the human disease state of “cold” can endanger human health.

However, this study has several limitations. First, it relied on a cross-sectional design and used qualitative and subjective indicators. Second, the KDC survey was provided to patients who had been admitted to a group of traditional Korean medicine clinics and hospitals in Korea, rather than to members of the general population. Third, survey respondents were assigned to the CHHF and non-CHHF groups based on the questionnaire answers, rather than a doctor's diagnostic findings. Fourth, although the questions on dyspepsia were for the most part based on the functional dyspepsia symptoms described in Rome II [12], a verified questionnaire on dyspepsia was not used. Additionally, the questions referring to digestion status were 2-point scales, questions referring to appetite were 4-point scales, and dyspepsia symptoms were 3-point scales. Therefore we could not sum the total scores for examination. Fifth, we also used self-reported survey responses to determine which patients had organic digestive diseases (and subsequently exclude the identified patients from our analysis). However, the presence or absence of organic disease was not verified based on doctors' examinations, such as endoscopic findings.

Therefore, we believe that a follow-up study is necessary to accurately define the relationship between CHHF and functional dyspepsia. Additional studies to delineate the mechanism between dyspepsia and CHHF are also needed. We hope that future studies will better reveal the precise correlation between these two symptoms and their underlying cause.

## 5. Conclusions

In this study, we were able to verify that patients with CHHF have more chronic (lasting more than 6 months) functional dyspepsia symptoms, especially bloating.

## Supplementary Material

Supplementary Table1: This table shows the entire questionnaires on cold hypersensitivity in the hands and feet (CHHF) and dyspepsia. The subjects were asked to answer the questionnaire based on their usual status within the past 6 months. The criteria for appetite responses were as follows: “1. Very good” refers to the desire to eat more foods despite satiety after meal; “2. Good” refers to the case in which one feels hungry at mealtimes and wants to eat food; “3. Average” refers to the case in which one eats meals at mealtimes, but does not have a good appetite; and “4. Not good” refers to the case in which one has no appetite at mealtimes and does not have a good sense of taste, even when eating. Furthermore, the criteria for responses regarding digestion symptoms were as follows: “1. Often” refers to greater than or equal to 2 times per week, “2. Sometimes” refers to greater than or equal to 3 times per month, and “3. Rarely” refers to less than or equal to 2 times per month.
Supplementary Figure 1: This figure shows odds ratios (ORs) for dyspepsia before and after propensity score matching, according to CHHF status. The ORs of responses (“Often” and “Sometimes”) were analyzed for each symptom, except for digestion status and appetite.

## Figures and Tables

**Table 1:** General characteristics of the study subjects.

Variable	Before matching			After matching		
	CHHF	Non-CHHF	*P* value	CHHF	Non-CHHF	*P* value
	(*n* = 1209)	(*n* = 1744)		(*n* = 640)	(*n* = 640)	
Sex						
Male	226 (18.7)	769 (44.1)	<0.001	168 (26.3)	173 (27)	0.752
Female	983 (81.3)	975 (55.9)		472 (73.8)	467 (73)	
Age (y)	44.6 ± 13.8	47.4 ± 14.8	<0.001	44.9 ± 14.8	45 ± 14.4	0.858
Height (cm)	161.3 ± 7.6	163.1 ± 8.8	<0.001	161.6 ± 8.1	161.2 ± 8.0	0.375
Weight (kg)	57.4 ± 8.8	64.2 ± 11.0	<0.001	59.8 ± 8.8	59.4 ± 9.1	0.447
BMI (kg/m^2^)	22 ± 2.8	24.1 ± 3.2	<0.001	22.9 ± 2.8	22.8 ± 2.8	0.731

Results are presented as *n* (%) or mean ± standard deviation.

CHHF: cold hypersensitivity in the hands and feet; Non-CHHF: noncold hypersensitivity in the hands and feet; BMI: body mass index.

**Table 2:** Dyspepsia in the CHHF and non-CHHF groups before and after propensity matching.

Variable	Before matching			After matching		
	CHHF	Non-CHHF	*P* value	CHHF	Non-CHHF	*P* value
	*n* (%)	*n* (%)		*n* (%)	*n* (%)	
Digestion						
Good	814 (67.3)	1453 (83.3)	<0.001	440 (68.8)	513 (80.2)	<0.001
Bad	395 (32.7)	291 (16.7)		200 (31.3)	127 (19.8)	
Appetite						
Extremely good	74 (6.1)	131 (7.5)	<0.001	43 (6.7)	45 (7.0)	0.045
Good	608 (50.3)	1014 (58.1)		319 (49.9)	366 (57.2)	
Average	423 (35)	508 (29.1)		223 (34.9)	188 (29.4)	
Not good	103 (8.5)	91 (5.2)		54 (8.5)	41 (6.4)	
Discomfort in the upper abdomen						
Often	74 (6.1)	40 (2.3)	<0.001	32 (5.0)	17 (2.7)	0.038
Sometimes	441 (36.5)	410 (23.5)		210 (32.8)	193 (30.2)	
Rarely	694 (57.4)	1294 (74.2)		398 (62.2)	430 (67.2)	
Vomiting						
Often	5 (0.4)	5 (0.3)	<0.001	3 (0.5)	3 (0.5)	0.094
Sometimes	133 (11)	98 (5.6)		67 (10.5)	45 (7.0)	
Rarely	1071 (88.6)	1641 (94.1)		570 (89.1)	592 (92.5)	
Motion sickness						
Often	40 (3.3)	19 (1.1)	<0.001	16 (2.5)	11 (1.7)	0.001
Sometimes	327 (27)	292 (16.7)		165 (25.8)	113 (17.7)	
Rarely	842 (69.6)	1433 (82.2)		459 (71.7)	516 (80.6)	
Exhaustion when hungry						
Often	132 (10.9)	120 (6.9)	<0.001	60 (9.4)	41 (6.4)	0.057
Sometimes	524 (43.3)	617 (35.4)		260 (40.6)	245 (38.3)	
Rarely	553 (45.7)	1007 (57.7)		320 (50)	354 (55.3)	
Belching						
Often	135 (11.2)	125 (7.2)	<0.001	70 (10.9)	53 (8.3)	0.272
Sometimes	408 (33.7)	578 (33.1)		217 (33.9)	224 (35.0)	
Rarely	666 (55.1)	1041 (59.7)		353 (55.2)	363 (56.7)	
Epigastric burning						
Often	48 (4.0)	41 (2.4)	<0.001	19 (3.0)	17 (2.7)	0.049
Sometimes	354 (29.3)	396 (22.7)		194 (30.3)	156 (24.4)	
Rarely	807 (66.7)	1307 (74.9)		427 (66.7)	467 (73.0)	
Postprandial fullness						
Often	73 (6.0)	47 (2.7)	<0.001	32 (5.0)	19 (3.0)	0.001
Sometimes	326 (27.0)	285 (16.3)		166 (25.9)	120 (18.8)	
Rarely	810 (67.0)	1412 (81.0)		442 (69.1)	501 (78.3)	
Nausea						
Often	32 (3.6)	20 (1.6)	<0.001	13 (2.8)	12 (2.6)	0.002
Sometimes	228 (25.6)	187 (15.2)		121 (25.7)	74 (16.2)	
Rarely	631 (70.8)	1024 (83.2)		336 (71.5)	370 (81.1)	
Epigastric pain (related to digestion)						
Often	40 (3.3)	22 (1.3)	<0.001	12 (1.9)	9 (1.4)	0.078
Sometimes	250 (20.7)	223 (12.8)		118 (18.4)	90 (14.1)	
Rarely	919 (76.0)	1499 (86.0)		510 (79.7)	541 (84.5)	
Bloating						
Often	76 (6.3)	51 (2.9)	<0.001	35 (5.5)	16 (2.5)	<0.001
Sometimes	422 (34.9)	419 (24)		228 (35.6)	157 (24.5)	
Rarely	711 (58.8)	1274 (73.1)		377 (58.9)	467 (73.0)	

*P* values are calculated from chi-square tests of the CHHF versus non-CHHF groups.

CHHF: cold hypersensitivity in the hands and feet; Non-CHHF: noncold hypersensitivity in the hands and feet.

Sample questions: digestion: “how is your digestion?”; appetite: “how is your appetite?”; symptoms (discomfort in the upper abdomen, vomiting, motion sickness, exhaustion when hungry, belching, epigastric burning, postprandial fullness, nausea, epigastric pain, and bloating): “do you have any of the following symptoms?”

**Table 3:** The odds ratios and 95% confidence intervals for dyspepsia before and after propensity matching according to CHHF status.

Variable	Non-CHHF	Before matching		After matching	
		CHHF OR (95% CI)	*P* value	CHHF OR (95% CI)	*P* value
Digestion: bad	Ref	2.423 (2.036–2.884)	<0.001	1.836 (1.421–2.372)	<0.001
Appetite: not good	Ref	1.693 (1.264–2.268)	<0.001	1.349 (0.885–2.056)	0.165
Discomfort in the upper abdomen	Ref	2.134 (1.825–2.495)	<0.001	1.245 (0.990–1.566)	0.061
Vomiting	Ref	2.053 (1.572–2.680)	<0.001	1.515 (1.031–2.226)	0.035
Motion sickness	Ref	2.008 (1.689–2.389)	<0.001	1.641 (1.264–2.130)	<0.001
Exhaustion when hungry	Ref	1.621 (1.398–1.879)	<0.001	1.238 (0.994–1.542)	0.057
Belching	Ref	1.207 (1.041–1.400)	0.013	1.065 (0.854–1.329)	0.573
Epigastric burning	Ref	1.490 (1.268–1.751)	<0.001	1.347 (1.060–1.711)	0.015
Postprandial fullness	Ref	2.095 (1.769–2.481)	<0.001	1.615 (1.255–2.077)	<0.001
Nausea	Ref	2.038 (1.656–2.509)	<0.001	1.716 (1.260–2.336)	0.001
Epigastric pain	Ref	1.931 (1.598–2.332)	<0.001	1.393 (1.044–1.858)	0.024
Bloating	Ref	1.899 (1.625–2.219)	<0.001	1.883 (1.489–2.382)	<0.001

CHHF: cold hypersensitivity in the hands and feet; Non-CHHF: noncold hypersensitivity in the hands and feet; OR: odds ratio; CI: confidence interval; Ref: reference.

“Non-CHHF” was employed as the reference in every analysis.

Sample questions: digestion: “how is your digestion?”; appetite: “how is your appetite?”; symptoms (discomfort in the upper abdomen, vomiting, motion sickness, exhaustion when hungry, belching, epigastric burning, postprandial fullness, nausea, epigastric pain, and bloating): “do you have any of the following symptoms?” Symptoms are the sum of “often” and “sometimes” responses.
