# Supplementary material for: Cold Hypersensitivity in the Hands and Feet May Be Associated with Functional Dyspepsia: Results of a Multicenter Survey Study
Source: Evid Based Complement Alternat Med. 2016 Mar 16;2016:8948690. doi: 10.1155/2016/8948690 (PMC4812350; doi:10.1155/2016/8948690)
Supplement: Supplementary file 1 — Supplementary Table1: This table shows the entire questionnaires on cold hypersensitivity in the hands and feet (CHHF) and dyspepsia. The subjects were asked to answer the questionnaire based on their usual status within the past 6 months. The criteria for appetite responses were as follows: “1. Very good” refers to the desire to eat more foods despite satiety after meal; “2. Good” refers to the case in which one feels hungry at mealtimes and wants to eat food; “3. Average” refers to the case in which one eats meals at mealtimes, but does not have a good appetite; and “4. Not good” refers to the case in which one has no appetite at mealtimes and does not have a good sense of taste, even when eating. Furthermore, the criteria for responses regarding digestion symptoms were as follows: “1. Often” refers to greater than or equal to 2 times per week, “2. Sometimes” refers to greater than or equal to 3 times per month, and “3. Rarely” refers to less than or equal to 2 times per month. Supplementary Figure 1: This figure shows odds ratios (ORs) for dyspepsia before and after propensity score matching, according to CHHF status. The ORs of responses (“Often” and “Sometimes”) were analyzed for each symptom, except for digestion status and appetite. [file 8948690.f1.pdf]

Supplementary Materials

Supplementary Table 1. Questionnaire about dyspepsia and CHHF

**Habits Questionnaire**				
Please answer the following on habits in the **past 6 months** based on your **usual lifestyle**. Ask the person in charge if you are unsure. You can **check more than once for questions marked by (*)**.				
**Heat and Cold**	Are your hands cold or warm?	1☐Warm 2☐Normal 3☐Cold 4☐Not sure		
	Are your feet cold or warm?	1☐Warm 2☐Normal 3☐Cold 4☐Not sure		
**Digestion**	How is your digestion? 1☐Good 2☐Bad			
	How is your appetite? 1☐Very good 2☐Good 3☐Average 4☐Not good			
	Do you have any of the following symptoms:			
	**Item**	**Often**	**Sometimes**	**Rarely**
	Discomfort in the upper abdomen	1☐	2☐	3☐
	Vomiting	1☐	2☐	3☐
	Motion sickness	1☐	2☐	3☐
	Exhaustion when hungry	1☐	2☐	3☐
	Belching	1☐	2☐	3☐
	Epigastric burning	1☐	2☐	3☐
	Postprandial fullness	1☐	2☐	3☐
	Nausea	1☐	2☐	3☐
	Epigastric pain (related to digestion)	1☐	2☐	3☐
	Bloating	1☐	2☐	3☐

Supplementary Figure 1. The odds ratios and 95% confidence intervals for dyspepsia before and after propensity matching according to CHHF status.

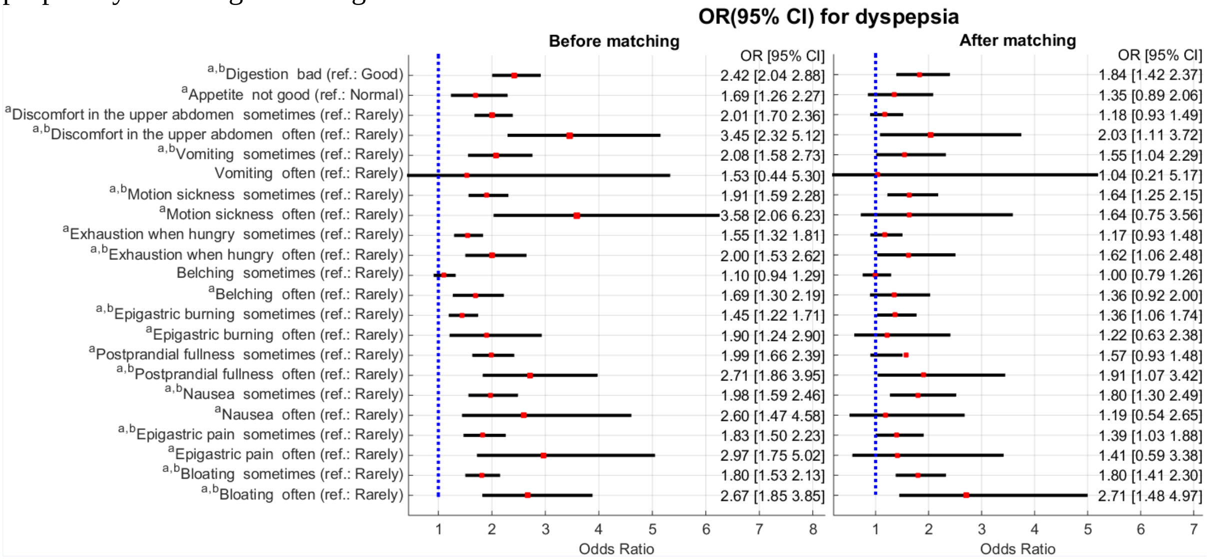

CHHF: cold hypersensitivity in the hands and feet; OR, odds ratio; CI, confidence interval; ref.: reference
"Normal" responses are the sum of "very good," "good," and "average" responses.
Sample questions: Digestion, "How is your digestion?" Appetite, "How is your appetite?" Symptoms (discomfort in the upper abdomen, vomiting, motion sickness, exhaustion when hungry, belching, epigastric burning, postprandial fullness, nausea, epigastric pain, and bloating), "Do you have any of the following symptoms?"
[a]$P < 0.05$ and statistically significant before matching; [b]$P < 0.05$ and statistically significant after matching.
